# Quantitative assessment data of PAHs and N-PAHs in core sediments from the Niger Delta, Nigeria

**DOI:** 10.1016/j.dib.2020.106484

**Published:** 2020-11-02

**Authors:** Ihuoma N. Anyanwu, Francis D. Sikoki, Kirk T. Semple

**Affiliations:** a Department of Biological Sciences, Alex Ekwueme Federal University Ndufu-Alike, P.M.B 1010, Abakaliki, Ebonyi State, Nigeria; b Lancaster Environment Centre, Lancaster University, Lancaster LA1 4YQ, United Kingdom; c Centre for Marine Pollution Monitoring and Seafood Safety, University of Port Harcourt, P.M.B 5323, Choba, Rivers State, Nigeria

**Keywords:** PAHs, N-PAHs, Sediment cores, Niger Delta, Nigeria

## Abstract

Polycyclic aromatic compounds (PACs) pollution has been the focus of environmental research, mostly due to their mutagenicity, carcinogenicity, teratogenicity and genotoxicity. Concentrations of polycyclic aromatic hydrocarbons (PAHs) and the nitrogen-containing analogues (N-PAHs) (which tend to accumulate in sediments rather than water) was measured in 2 cm intervals segments from Bonny Estuary, Niger Delta using GC–MS. Data showed that PAHs/N-PAHs levels ranged from 8699 to 22,528 µg/kg and 503–2020 µg/kg, respectively. Furthermore, the data revealed that ƩPAHs level in the estuarine segments was > 45% higher than DPR/EGASPIN intervention limit. This gives insight on PAHs/N-PAHs contamination in the oil rich region.

## Specifications Table

**Table:** 

Subject area	Environmental Science
More specific subject area	Pollution
Type of data	Figures, Tables.
How data was acquired	GC–MS analysis (Thermo Trace GC Ultra- DSQ).
Data format	Analysed, Raw.
Parameters of Data Collection	Survey of PAHs and N-PAHs in core sediments from Bonny Estuary, Niger Delta.
Description of Data Collection	Sediment samples were collected from 3 stations using Uwitec manufactured Plexiglas's tubes mounted on a triple sediment corer type 90 mm.
Data source location	Niger Delta, Nigeria
Data accessibility	Data available in the article
Related research article	I.N. Anyanwu, F.D. Sikoki, K.T. Semple. Risk assessment of PAHs and N-PAH analogues in sediment cores from the Niger Delta, Mar. Pollut. Bull. (2020). https://doi.org/10.1016/j.marpolbul.2020.111684.

## Value of Data

• Data provides insight on PAHs/N-PAHs that exists in the Niger Delta environment.
• The data could be useful to environmental scientists, toxicologists, limnologists and policy makers.
• Data can be compared with other scientific manuscripts and/or be useful in future monitoring of sediment recovery.
• Data revealed extent of PAHs/N-PAHs pollution and underground water contamination in the oil rich region.

## 1 Data Description

**Fig. 1:**
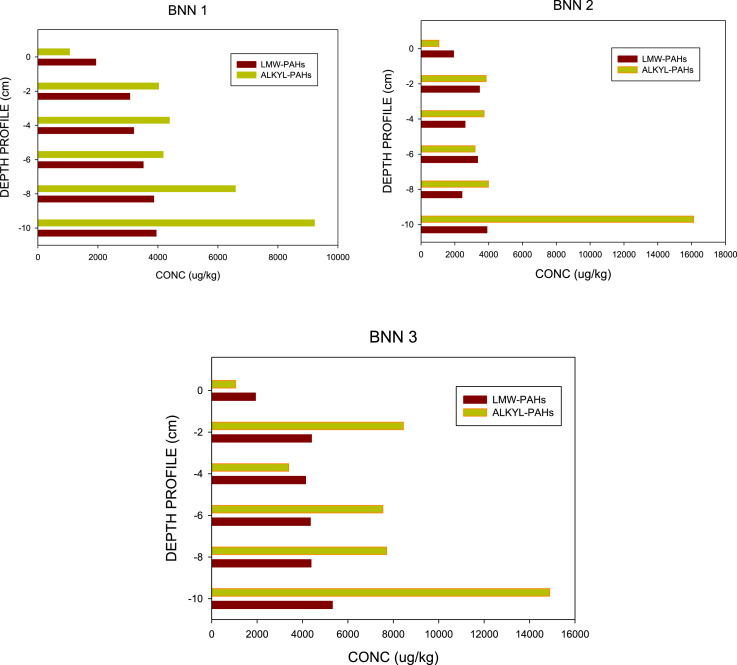
LMW–ALKYL PAHs ranges (μg/kg) in core segments from Bonny Estuary, Niger Delta. Conc = concentration; LMW = Low Molecular Weight; ALKYL = Alkylated PAHs; BNN 1 = location 1; BNN 2 = location 2; BNN 3 = location 3. Y-axis depicts: 0 = control; –2 = 0–2 cm; –4 = 2–4 cm; –6 = 4–6 cm; –8 = 6–8 cm; –10 = 8–10 cm.

**Fig. 2:**
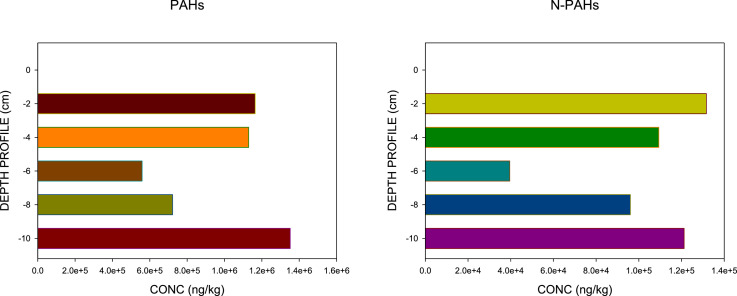
Temporal flux of PAHs and N-PAHs (ng/cm^2^/yr) in core segments from Bonny Estuary, Niger Delta. Y-axis depicts: 0 = control; –2 = 0–2 cm; –4 = 2–4 cm; –6 = 4–6 cm; –8 = 6–8 cm; –10 = 8–10 cm.

PAHs and N-PAHs are known to co-existence in contaminates sites, however, environmental analysis have focused majorly on PAHs [1], [2], [3], [4]. The data reported in this article is derived from sediment survey of PAHs and N-PAHs in Bonny Estuary, Niger Delta. The compiled GC–MS data analysis are included as Supplemental Material. In this study, Fig. 1 displays the LMW–ALKYL PAHs ranges (μg/kg) in core segments from Bonny Estuary, Niger Delta. The plot shows that low molecular weight PAHs contributed > 50% ƩPAHs measured in the estuarine sediments, with alkyl–PAHs recording high concentrations, while high molecular weight N-PAHs recorded > 60% N-PAHs measured in the estuary [5]. Also, underground well (used as control) recorded elevated concentrations of PAHs/N-PAHs [5]. Fig. 2 portrays the temporal flux of PAHs and N-PAHs (ng/cm^2^/yr) in core segments from Bonny Estuary, Niger Delta. The calculated flux revealed that low PAHs/N-PAHs load occurred in the 50 s and early 70 s; and high deposition in early 90 s to 2000s [5], [6], [7], [8]. Also, Table 1 describes the coordinates of sampling locations. The description of sampling locations is provided in Anyanwu et al. [5]. Furthermore, analytes, abbreviations, chemical formula, chemical structure and molecular mass; as well as; analyte list, abbreviations and detection limits are shown in Tables 2 and 3, respectively. The supplementary material provides the raw data relative to each individual repeat used to calculate the average, standard deviations and standard errors for Tables 4 and 5. The Tables which highlights the profiles of PAHs and N-PAHs in segment core samples from the Niger Delta, shows that ƩPAHs measured in the Estuary ranged from 16,635 µg/kg (0–2 cm) to 22,528 µg/kg (8–10 cm) (Table 4) and ƩN-PAHs ranged from 503 – 2020 µg/kg with B[a]A, dibenz-acridines and B[h]Q recording elevated values (Table 5). The mean concentrations of the measured chemicals and their toxic ratios are also recorded in Anyanwu et al. [5]. Interestingly, the data revealed that ƩPAHs concentrations in the estuary was > 45% higher than DPR/EGASPIN intervention limit [5].

**Table 1:** Description and coordinates of sampling locations.

Location	Description	Coordinates	No of segment
BNN1	Station with no ongoing activity	N 4°46′33.73′'E 7°00′.18.85′'	5
BNN2	Port Harcourt Harbour (shipping)	N 4°46′02.43′' E 7°00′.10.56′'	5
BNN3	Area of Cement Bagging Factory	N 4°45′71.13′' E 7°00′05.01′'	5

**Table 2:** Analytes, abbreviations, chemical formula, chemical structure and molecular mass.

Analyte(s) (PAHs)	Chem. formula	Chem. Structure	Mol. mass	Analyte(s)	Chem. formula	Chem. structure	Mol. mass
Naphthalene^⁎⁎^ (N0)	C_10_H_8_		128.1	Benzo[k]fluoranthene^⁎⁎^ (B[f]F)	C_20_H_12_		252.3
2-methyl-naphthalene (N1)	C_11_H_10_		142.2	Benzo[e]pyrene (B[e]P)	C_20_H_12_		252.3
1-methyl-naphthalene (N2)	C_11_H_10_		142.2	Benzo[a]pyrene^⁎⁎^ (B[a]P)	C_20_H_12_		252.3
Biphenyl (Bph)	C_12_H_10_		154.2	Perylene (Per)	C_20_H_12_		252.3
2,6-dimethyl-naphthalene (N3)	C_12_H_12_		156.2	Dibenz[ah] anthracene^⁎⁎^ (D[ah]A)	C_22_H_14_		278.3
Acenaphthylene^⁎⁎^ (Acl)	C_12_H_8_		152.2	Indeno[123-cd] pyrene^⁎⁎^ (ID)	C_22_H_12_		276.3
Acenphthene ^⁎⁎^ (Acc)	C_12_H_10_		154.2	Benzo[ghi]perylene^⁎⁎^ (B[ghi]P)	C_22_H_12_		276.3
2,3,6-trimethyl naphthalene (N4)	C_13_H_14_		170.2	**N-PAHs**			
Fluorene^⁎⁎^ (F0)	C_13_H_10_		166.2	Quinoline٭ (Quin.)	C_9_H_7_N		129.1
Phenanthrene^⁎⁎^ (P0)	C_14_H_10_		178.2	Isoquinoline (Isoquin.)	C_9_H_7_N		129.1
Anthracene^⁎⁎^ (AN)	C_14_H_10_		178.2	Benzo[h]quinoline٭ (B[h]Q)	C_13_H_9_N		179.2
1 methyl phenanthrene (P1)	C_15_ H_12_		192.2	1,7-Phenanthroline (1,7-Phen)	C_12_H_8_N_2_		180.2
Flouranthene^⁎⁎^ (FL)	C_16_H_10_		202.2	4,7-Phenanthroline (4,7-Phen)	C_12_H_8_N_2_		180.2
Pyrene^⁎⁎^ (PY)	C_16_H_10_		202.2	Benzo[a]acridine٭ (B[a]A)	C_17_H_11_N		229.2
Benzo[a] anthracene^⁎⁎^ (B[aA)	C_18_H_12_		228.2	Dibenz[a,h]acridine٭ (D[ah]A)	C_21_H_13_N		279.3
Chrysene^⁎⁎^ (C0)	C_18_H_12_		228.2	Dibenz[c,h]acridine٭ (D[ch]A)	C_21_H_13_N		279.3
Benzo[b] fluoranthene^⁎⁎^ (B[b]F)	C_20_H_12_		252.3				

16 EPA PAHs (^⁎⁎^); carcinogenic N-PAHs (٭).

**Table 3:** Analyte list, abbreviations and detection limits.

PAHs	Abbreviation	IDL (ng/ml)	MDL (µg/kg)
Naphthalene	N0	0.1	25.7
2-methyl-naphthalene	N2	0.1	3.8
1-methyl-naphthalene	N1	0.3	ND
Biphenyl	Bph	0.5	23.7
2,6-dimethylnaphthalene	N3	0.9	46.2
Acenaphthylene	Acl	1.9	33.8
Acenaphthene	Ace	0.5	42.4
2,3,6-trimethyl-naphthalene	N4	1.5	48.4
Flourene	F0	1.3	32.3
Phenanthrene	P0	0.7	29.3
Anthracene	AN	0.2	41.1
1-methyl-phenanthrene	P1	2.5	23.3
Flouranthene	FL	0.1	24.5
Pyrene	PY	0.6	27.4
Benzo[a]anthracene	B[a]A	0.2	34.6
Chrysene	C0	0.1	27.9
Benzo[b]flouranthene	B[b]F	0.1	53.2
Benzo[k]flouranthene	B[k]F	0.2	67.8
Benzo[e]pyrene	B[e]P	0.1	20.7
Benzo[a]pyrene	B[a]P	0.2	24.3
Perylene	Per	0.2	36.1
Indeno[123-cd]pyrene	ID	0.5	19.6
Dibenz[ah]anthracene	D[h]A	1.5	11.8
Benzo[ghi]perylene	B[ghi]P	0.5	13.6
N-PAHs			
Quinoline	Quin	0.8	48.6
Isoquinoline	Isoquin	0.7	39.7
B[h]quinoline	B[h]Q	5.9	61.5
1,7-phenanthroline	1,7-Phen	1.9	54.3
4,7-phenanthroline	4,7-Phen	2.9	70.3
Benzo[a]acridine	B[a]A	0.2	82.2
Dibenz[ah]acridine	D[ah]A	0.1	75.3
Dibenz[ch]acridine	D[ch]A	0.2	76.1

IDL = instrument detection limit; MDL = method detection limit

**Table 4:** Profiles of PAHs in segment core samples from the Niger Delta.

	Control (µg/kg)	BNN 1 (µg/kg)					BNN 2 (µg/kg)					BNN 3 (µg/kg)				
PAHs	0 cm	0–2 cm	2–4 cm	4–6 cm	6–8 cm	8–10 cm	0–2 cm	2–4 cm	4–6 cm	6–8 cm	8–10 cm	0–2 cm	2–4 cm	4–6 cm	6–8 cm	8–10 cm
N0	157.6 ± 3.2	1085.4 ± 63.2	1251.6 ± 77.2	1155.8 ±128.1	1155.8 ± 47.0	1322.2 ± 11.4	1140.8 ± 47.1	668.4± 7.9	758.0± 28.9	674.8 ± 44.1	671.1 ± 56.8	760.7 ± 78.9	1304.4 ± 90.0	1129.6 ± 15.6	1205.0 ± 10.3	1475.7 ± 71.1
N2	7.3 ± 0.0	408.6 ± 12.7	478.7 ± 9.8	486.1 ± 13.8	596.6 ± 19.7	695.1 ± 14.0	300.7 ± 1.9	282.6± 3.2	267.9 ± 29.4	320.31 ± 22.5	912.69 ± 86.7	210.6 ± 11.6	301.2 ± 5.6	835.3 ± 38.1	993.9 ± 7.9	1410.5 ± 46.1
N1	64.9 ± 0.9	234.5 ± 14.4	339.1 ± 15.1	327.9 ± 25.9	345.8 ± 17.9	445.1 ± 14.1	485.2 ± 22.8	461.4 ± 8.9	490.5 ± 15.4	550.2 ± 9.8	1406.6 ± 12.3	487.2 ± 30.9	586.3 ± 44.4	1386.0 ± 12.4	1480.4 ± 69.5	2178.9 ± 22.6
Bph	46.5 ± 0.1	312.1 ± 2.9	373.8 ± 25.1	384.4 ± 24.9	466.0 ± 30.0	518.1 ± 17.8	285.9 ± 18.2	253.9± 4.8	372.5 ± 9.6	209.5 ± 15.5	442.8 ±48.6	140.8 ± 1.2	223.5 ± 12.4	272.2 ± 26.9	265.8 ± 18.1	442.1 ± 2.9
N3	220.9 ± 0.3	1532.7 ± 33.3	1665.4 ± 57.9	1579.3 ± 71.5	3460.4 ± 50.1	5463.9 ± 99.0	2031.8 ± 16.6	2118.2 ± 41.9	1320.3 ±48.7	2281.1 ± 78.9	10,069.1 ± 17.6	1370.0 ± 2.2	1289.8 ± 19.5	4064.1 ± 150.7	3748.4 ± 66.4	8148.5 ± 156.7
Acl	42.1 ± 0.1	76.8 ± 0.6	91.3 ± 3.9	86.7 ± 2.7	99.6 ± 4.5	99.6 ± 1.0	55.8 ± 3.1	67.8 ± 6.1	167.5 ± 63.9	62.7 ± 3.6	104.7 ± 0.4	174.5 ± 3.9	93.4 ± 1.9	113.9 ± 4.9	159.4 ± 5.4	247.8 ± 7.7
Acc	108.9± 0.8	340.0 ± 2.4	431.1 ± 41.4	462.8 ± 19.6	573.4 ± 22.2	586.5 ± 2.6	374.6 ± 24.2	345.1 ± 2.7	381.1 ± 20.1	283.3 ± 10.1	523.6 ± 36.8	281.1 ± 36.8	378.5 ± 26.5	418.3 ± 35.2	275.1 ± 150.8	848.2 ± 34.8
N4	736.3 ± 1.0	1218.5 ± 65.1	1314.0 ± 1.0	1181.8 ± 29.4	1611.6 ± 55.2	2080.2 ± 69.5	587.6 ± 17.9	587.8 ± 22.9	619.0 ± 49.4	522.7 ± 74.8	3199.7 ± 281.3	5721.7 ± 171.5	663.9 ± 16.9	811.3 ± 62.5	1166.2 ± 64.3	2731.5 ± 54.2
F0	228.8 ± 3.6	356.3 ± 8.6	339.2 ± 23.4	433.2 ± 11.7	569.8 ± 19.1	541.3 ± 30.4	396.5 ± 25.8	346.2 ± 17.1	414.7 ± 1.3	326.3 ± 10.1	904.4 ± 9.6	483.2 ± 2.4	574.9 ± 11.9	630.8 ± 37.9	752.1 ± 2.2	126.3 ± 19.1
P0	1232.4 ± 4.1	603.6 ± 6.0	409.3 ± 0.7	655.1 ± 16.0	665.2 ± 2.0	566.5 ± 43.3	996.6 ± 19.8	733.0 ± 13.2	974.7 ± 9.2	667.6 ± 24.1	1085.1± 21.9	2347.9 ± 141.6	1409.9 ± 23.2	1696.0 ± 12.2	1546.2 ± 42.1	1997.3 ± 105.0
AN	151.4 ± 0.7	288.7 ± 15.2	297.4 ± 0.1	334.3 ± 16.1	333.7 ± 2.6	308.7± 17.1	206.6 ± 5.8	192.1 ± 15.7	279.9 ± 7.6	193.7 ± 1.4	170.4 ± 0.8	210.7 ± 1.1	152.3 ± 21.1	88.2 ± 2.8	174.6 ± 1.0	180.5 ± 13.8
P1	151.5 ± 0.3	629.4 ± 51.9	586.2 ± 5.2	601.8 ± 2.8	570.1 ± 11.7	533.0 ± 2.0	446.9 ± 46.9	279.3 ± 4.9	500.1 ± 22.6	316.0 ± 5.9	537.4 ± 15.9	651.6 ± 95.8	546.8 ± 23.7	443.4 ± 9.6	318.6 ± 15.9	417.8 ± 1.9
FL	331.4 ± 0.5	525.3 ± 6.4	521.9 ± 30.2	578.2 ± 62.4	537.8 ± 9.8	568.8 ± 1.0	386.3 ± 37.4	481.1 ± 17.1	1030.9 ± 56.3	312.8 ± 13.0	339.2 ± 31.9	415.5 ± 47.1	277.0 ± 0.8	245.7 ± 9.9	216.9 ± 35.2	229.9 ± 20.9
PY	295.6 ± 0.7	731.1 ± 18.5	731.7 ± 14.1	820.7 ± 38.5	764.7 ± 10.6	813.1 ± 11.4	553.1 ± 49.6	671.4 ± 15.4	1324.3 ± 43.0	475.8 ± 20.9	521.4 ± 35.6	516.1 ± 55.2	473.8 ± 2.5	314.9 ± 0.7	241.4 ± 22.6	304.3 ± 60.9
B[a]A	38.9 ± 0.5	817.6 ± 52.7	866.6 ± 27.2	1200.1 ± 22.7	836.5 ± 24.4	964.5 ± 5.5	508.6 ± 84.7	1091.1 ± 34.7	1711.2 ± 26.8	424.6 ± 15.7	582.2 ± 36.7	399.9 ± 44.1	372.8 ± 2.7	213.6 ± 1.1	198.2 ± 7.7	233.8 ± 7.7
C0	56.9 ± 0.1	795.3 ± 65.7	784.9 ± 11.7	1069.5 ± 60.3	785.3 ± 31.8	905.0 ± 5.4	598.7 ± 41.4	144.1 ± 17.7	275.9 ± 20.9	63.9 ± 9.4	88.4 ± 2.4	355.7 ± 34.6	331.27 ± 10.7	192.1 ± 3.6	171.6 ± 1.9	194.9 ± 4.5
B[b]F	336.6 ± 1.4	1568.7 ± 113.4	1623.1 ± 76.7	2115.6 ± 48.1	1532.4 ± 22.7	1663.8 ± 52.1	336.5 ± 33.4	204.3 ± 12.6	1470.7 ± 52.1	298.5 ± 10.8	127.9 ± 4.1	1189.9 ± 65.6	741.0 ± 30.3	697.1 ± 1.8	884.7 ± 44.6	527.2 ± 1.1
B[k]F	410.1 ± 0.1	1990.8 ± 14.8	2000.7 ± 15.3	2485.2 ± 4.3	1767.4 ± 13.2	2021.5 ± 46.0	221.2 ± 81.2	673.5 ± 68.9	1068.7 ± 42.3	303.9 ± 57.9	483.3 ± 87.5	304.3 ± 23.2	241.1 ± 2.9	156.0 ± 0.2	195.8 ± 0.6	279.4 ± 15.0
B[e]P	35.8 ± 0.1	158.3 ± 10.0	173.5 ± 0.1	188.6 ± 5.1	160.7 ± 3.1	164.9 ± 4.1	120.4 ± 2.2	129.9 ± 1.5	175.5 ± 13.7	83.6 ± 1.5	127.9 ± 5.2	35.1 ± 3.5	45.0 ± 0.1	0.0 ± 0.0	67.2 ± 0.6	68.7 ± 3.9
B[a]P	59.5 ± 0.1	113.6 ± 3.9	123.4 ± 4.8	190.1 ± 1.1	120.1 ± 3.5	127.7 ± 0.8	130.7 ± 6.6	206.7 ± 13.5	386.6 ± 10.7	134.2 ± 4.7	128.2 ± 1.7	343.5 ± 43.6	101.8 ± 1.2	160.1 ± 1.6	216.5 ± 23.7	243.1 ± 5.9
Per	25.7 ± 0.1	ND	ND	ND	ND	ND	ND	ND	ND	ND	ND	ND	ND	ND	ND	ND
D[ah]A	10.2 ± 0.2	51.9± 1.1	32.2 ± 0.2	51.2 ± 2.2	49.8 ± 1.0	39.5 ± 4.5	31.1 ± 0.8	34.1 ± 0.4	109.4 ± 0.4	26.7 ± 1.1	31.6 ± 0.7	64.5 ± 1.3	32.8 ± 0.1	26.1 ± 0.6	54.0 ± 0.4	79.8 ± 0.8
ID	43.6 ± 0.1	219.7 ± 11.4	258.4 ± 19.3	311.4 ± 25.1	244.1 ± 2.4	246.3 ± 1.2	132.6 ± 3.9	203.8 ± 1.7	294.0 ± 6.9	80.2 ± 6.1	111.5 ± 3.4	99.2 ± 1.6	72.9 ± 1.8	42.7 ± 1.5	65.8 ± 0.6	94.0 ± 2.4
B[ghi]P	39.1 ± 0.1	301.1 ± 5.5	332.3 ± 1.5	363.1 ± 3.9	285.4 ± 4.7	306.8 ± 8.1	130.0 ± 4.1	249.0 ± 46.0	275.5 ± 3.5	87.1 ± 0.4	125.6 ± 3.1	72.2 ± 0.7	53.7 ± 3.9	27.7 ± 0.5	39.2 ± 1.6	68.6 ± 1.9
∑PAHs (µg/kg)	**4824.7**	**14,360.0**	**15,025.8**	**17,062.9**	**17,532.2**	**20,982.1**	**10,458.2**	**10,424.8**	**14,668.9**	**8699.5**	**22,694.8**	**16,635.9**	**10,268.1**	**13,965.1**	**14,437.0**	**22,528.8**
∑PAHs (mg/kg)	**4.8**	**85.0**					**67.0**					**77.8**				
DPR / EGASPIN Interv. Limit (mg/kg)	**40.0**	**40.0**					**40.0**					**40.0**				

Values = mean ± SE.

ND = PAHs not detected during analysis.

DPR = Department of Petroleum Resources.

EGASPIN = Environmental Guidelines and Standards for the Petroleum Industry in Nigeria.

**Table 5:** Profiles of N-PAHs in segment core samples from the Niger Delta.

	Control (µg/kg)	BNN 1 (µg/kg)					BNN 2 (µg/kg)					BNN 3 (µg/kg)				
N-PAHs	0 cm	0–2 cm	2–4 cm	4–6 cm	6–8 cm	8–10 cm	0–2 cm	2–4 cm	4–6 cm	6–8 cm	8–10 cm	0–2 cm	2–4 cm	4–6 cm	6–8 cm	8–10 cm
Quin*	43.8 ± 0.0	49.0 ± 0.1	49.7 ± 0.10	49.8 ± 0.4	50.3 ± 0.1	51.4 ± 0.5	54.5 ± 4.7	50.7 ± 1.1	168.6 ± 70.5	68.3 ± 19.4	49.8 ± 0.1	167.5 ± 70.8	96.4 ± 0.3	96.9 ± 0.1	167.7 ± 70.5	167.6 ± 71.2
Isoquin	34.6 ± 0.1	39.3 ± 0.0	39.5 ± 0.0	39.3 ± 0.2	39.7 ± 0.3	39.8 ± 0.1	42.9 ± 3.4	40.4 ± 1.0	136.7 ± 58.5	55.2 ± 15.8	39.4 ± 0.1	136.5 ± 58.6	78.2 ± 0.1	78.4 ± 0.0	136.8 ± 58.5	137.1 ± 58.8
B[h]Q*	63.3 ± 0.3	93.1 ± 1.0	95.6 ± 1.7	101.3 ± 0.5	104.1 ± 2.0	96.8 ± 1.0	76.5 ± 6.5	70.6 ± 2.0	221.5 ± 90.8	95.0 ± 24.0	67.1 ± 0.0	219.0 ± 90.8	128.9 ± 0.8	124.2 ± 0.2	214.4 ± 90.5	213.4 ± 90.8
1,7-Phen	43.9 ± 0.4	51.0 ± 0.0	50.6 ± 0.1	50.2 ± 0.9	56.3 ± 0.1	52.5 ± 0.6	57.6 ± 4.7	54.0 ± 1.0	185.2 ± 79.1	73.9 ± 21.8	53.0 ± 0.2	184.9 ± 78.8	105.8 ± 0.1	106.7 ± 0.3	186.5 ± 79.7	187.7 ± 80.3
4,7-Phen	65.6 ± 0.1	81.8 ± 0.0	81.7 ± 0.6	86.5 ± 5.9	82.7 ± 2.0	83.6 ± 0.3	77.4 ± 5.8	72.9 ± 1.6	241.1 ± 101.5	99.0 ± 27.9	72.4 ± 0.1	242.6 ± 103.2	139.2 ± 0.0	138.6 ± 0.1	241.8 ± 102.6	241.4 ± 103.9
B[a]A*	75.7 ± 0.6	93.8 ± 3.0	101.3 ± 1.0	98.3 ± 2.	99.4 ± 0.6	98.0 ± 1.3	88.3 ± 7.5	85.1 ± 1.9	330.0 ± 67.8	114.1 ± 31.8	81.5 ± 0.2	329.2 ± 69′8	160.9 ± 0.2	159.2 ± 0.1	376.8 ± 18.6	376.3 ± 18.7
D[ah]A*	68.1 ± 1.2	72.9 ± 0.0	72.8 ± 0.1	77.9 ± 0.0	72.9 ± 0.0	73.0 ± 0.3	85.3 ± 7.5	75.3 ± 1.6	304.4 ± 58.8	101.9 ± 29.6	72.4 ± 0.0	303.1 ± 57.6	144.7 ± 0.0	145.1 ± 0.2	302.2 ± 57.8	352.4 ± 8.2
D[ch]A*	66.9 ± 0.4	72.7 ± 0.6	72.5 ± 0.3	77. ± 0.7	72.3 ± 0.7	71.9 ± 0.3	80.5 ± 5.2	74.5 ± 1.8	297.9 ± 53.9	100.0 ± 28.0	72.6 ± 1.8	298.0 ± 56.2	140.4 ± 0.1	140.2 ± 0.0	295.0 ± 55.0	344.9 ± 5.0
∑N-PAHs (µg/kg)	**461.9**	**553.6**	**563.7**	**503.3**	**577.7**	**567.0**	**563.0**	**523.5**	**1885.4**	**707.4**	**508.2**	**1880.8**	**994.5**	**989.3**	**1921.2**	**2020.8**
∑NPAHs (mg/kg)	**0.5**	**2.8**					**4.2**					**7.8**				

٭ = carcinogenic N-PAHs (IARC, 2012; 2013). Values = mean ± SE.

## 2 Experimental Design, Materials and Methods

### 2.1 Sample collection

Sample collection was as described in Anyanwu et al. [5]. In brief, sediment cores (10 cm long) were collected from 3 stations in Bonny Estuary using Uwitec manufactured Plexiglas's tubes mounted on a triple sediment corer (Table 1), and sliced into 2 cm layers. Due to high pollution in the estuary and the surrounding waters, sediment sample was collected from an underground community well (drinking well) in the area to serve as control. Following collection and segmentation, samples were taken to the laboratory, oven dried at 50 °C, homogenised, sieved with 2 mm mesh size, stored in a container and transported to United Kingdom, where they were stored at 4 °C until analysis [5].

### 2.2 Chemicals

Chemical standards (PAH and N-PAH) were purchased from Thames Restek and Sigma-Aldrich, UK, respectively. Internal standard D_9_-acridine was purchased from Cambridge Isotopes Laboratories, UK. HPLC grade acetonitrile, methanol and ethyl-acetate were used for the analysis. Calibration curves were performed at ten levels ranging from 2.5 to 2500 ng/ml for PAHs and six ranging from100 to 2000 ng/ml for N-PAHs in ethyl acetate. Accepted linearity was obtained in all calibrations (r^2^ >0.99). The measured compounds are listed in Tables 2 and 3 [5].

### 2.3 Extraction procedure and GC–MS analysis/quantification

Sample extractions and GC–MS quantification was as reported in Anyanwu et al. [5]. In brief, 1–2 g sediments, mixed with 2 g anhydrous sodium sulphate NaSO_4_, was weighed into pre-conditioned extraction thimble (after conditioning for 4 h) and extracted in a Soxhlet device for 18 h using 300 ml solvent mixture of ACN/MeOH (8:2). Extract was concentrated to 1 ml (Büchi Rotavap R-144). Clean-up was performed over a 5 mm glass column containing 6 g of 2% water deactivated aluminium-oxide, topped with 1 g NaSO_4_ (all baked overnight at 450 °C) [1, 4]. The column was conditioned with 50 ml ACN/MeOH (8:2) and elution was with 50 ml ACN/MeOH (8:2). Elutes were rotary evaporated, solvent exchanged with ethyl-acetate and concentrate to 1 ml. Internal standard (D_9_-acridine) was added and samples were stored in the freezer until analysis with GC–MS. GC–MS analysis was performed with Thermo Trace GC Ultra- DSQ. ZB–Semi-Volatile column 30 m ˟ 0.25 mm ˟ 0.25 µm (Phenomenex, USA) was used. Scan acquisition was performed by selected ion monitoring (SIM) [5]. Data obtained from GC–MS analysis was used to derive the figures. The LMW PAHs/N-PAHs are 2- to 3-rings while, HMW group are 4- to 6-rings. Temporal flux was calculated according to Zaborska [7].

## Authors contributions

FDS: Sample collection. INA: Methodology, Laboratory analysis, Writing original draft, Review and Editing. KTS: Supervision, Review and Editing of first draft.

## Declaration of Competing Interest

The authors declare that they have no known competing interests.

## Appendix Supplementary materials

Image, application 1

## References

[1] Anyanwu I.N., Semple K.T. (2015). Biodegradation of phenanthrene-nitrogen-containing analogues in soil. Water Air Soil Pollut..

[2] Anyanwu I.N., Semple K.T. (2015). Phytotoxicity of phenanthrene and its nitrogen polycyclic aromatic hydrocarbon analogues in ageing soil. Water Air Soil Pollut..

[3] Anyanwu I.N., Clifford O.I., Semple K.T. (2017). Effects of single, binary and quinary mixture of phenanthrene and its N-PAHs on *Eisenia fetida* in soil. Water Air Soil Pollut..

[4] Anyanwu I.N., Semple K.T. (2016). Effects of phenanthrene and its nitrogen-containing analogues aged in soil on earthworm *Eisenia fetida*. Appl. Soil Ecol..

[5] Anyanwu I.N., Sikoki F.D., Semple K.T. (2020). Risk assessment of PAHs and N-PAH analogues in sediment cores from the Niger Delta. Mar. Pollut. Bull..

[6] UNEP (2011). Environmental Assessment of Ogoniland. http://www.unep.org/nigeria.

[7] Zaborska A. (2014). Anthropogenic lead concentrations and sources in Baltic Sea sediments based on lead isotopic composition. Mar. Pollut. Bull..

[8] Omokheyeke O., Sikoki F., Abdelmourhit L. (Aug 2015). Applying sediment cores and nuclear techniques for pollution assessment in the Bonny/new Calabar River Estuary, Niger Delta, Nigeria. Poster presented at the 23rd WiN Global Annual Conference: Women in Nuclear meet Atoms for Peace Programme and Women in Nuclear Global, c/o World Nuclear Association Tower House, London WC2E 7HA (United Kingdom); International Atomic Energy Agency.

